# Low Daily Dietary Sodium Intake is Associated with Faster Late‐Life Cognitive Decline in Community‐Dwelling Older Adults

**DOI:** 10.1002/alz70860_103286

**Published:** 2025-12-23

**Authors:** Han Tong, Ana W. Capuano, Puja Agarwal, David A. A. Bennett, Zoe Arvanitakis

**Affiliations:** ^1^ Rush University Medical Center, Chicago, IL, USA

## Abstract

**Background:**

Previous cross‐sectional studies have linked dietary sodium intake to cognitive function, though findings have been inconsistent. This study aims to examine the associations between daily sodium intake and late‐life cognitive decline in community‐dwelling older adults.

**Method:**

We studied 1520 individuals without dementia at baseline (75.1% female, mean (SD) age at baseline=80.7 ± 7.2 years) from the Rush Memory and Aging Project, a community‐based cohort study of aging. Participants underwent annual clinical evaluations with comprehensive neuropsychological testing and a food frequency questionnaire, from which daily dietary sodium intakes were estimated. Linear mixed‐effect models adjusting for age, sex, education, were used to examine the associations of calorie‐adjusted baseline daily dietary sodium intake in quintiles with subsequent cognitive decline (mean follow‐up = 7.6 years).

**Result:**

Compared to the 3^rd^ quintile group (reference group, median [IQR]=2264[2209‐2320] mg/day, similar to the 2,300 mg/day sodium intake limit recommended by multiple guidelines), the lowest quintile of sodium intake (1764[1609‐1861] mg/day) was associated with a faster decline in global cognition (estimate=‐0.023, SE=0.009, *p* = 0.009). The association remained significant after additionally adjusting (one variable at a time) for BMI, systolic and diastolic blood pressures, physical activity, total calorie intake, MIND diet score, history of hypertension, antihypertensive/diuretic use, history of diabetes, history of stroke, history of heart attack, history of smoking, serum sodium level. However, the association was no longer significant after additionally adjusting for the presence of any *APOE‐ε4* allele (estimate = ‐0.017, SE=0.009, *p* = 0.067). In addition, we found associations between the lowest quintile of sodium intake and faster declines in episodic memory, semantic memory, and perceptual speed, and between the 2nd lowest quintile of sodium intake (2064[2006‐2116] mg/day) and faster declines in perceptual speed, and semantic memory (all *p* <0.05). No other significant associations of low or high quintile groups with cognitive decline were identified (all *p* >0.05).

**Conclusion:**

In our study of more than 1500 older persons followed longitudinally, a low dietary sodium intake, but not high sodium intake, was associated with a faster decline in cognitive function, especially in memory and perceptual speed. Future research is needed to explore the relation of sodium intake to neuropathologies of dementia.

